# Biology of *Plasmodium falciparum* gametocyte sex ratio and implications in malaria parasite transmission

**DOI:** 10.1186/s12936-019-2707-0

**Published:** 2019-03-12

**Authors:** Noëlie Béré Henry, Samuel Sindié Sermé, Giulia Siciliano, Salif Sombié, Amidou Diarra, N’fale Sagnon, Alfred S. Traoré, Sodiomon Bienvenu Sirima, Issiaka Soulama, Pietro Alano

**Affiliations:** 1 Centre National de Recherche et de Formation sur le Paludisme, Ouagadougou, Burkina Faso; 2 Dipartimento di Malattie Infettive, Istituto Superiore di Sanità, Rome, Italy; 3 University Joseph Ki Zerbo, Ouagadougou, Burkina Faso; 4 Groupe de Recherche Action Santé, Ouagadougou, Burkina Faso

**Keywords:** Malaria, *Plasmodium falciparum*, Gametocyte, Transmission, Sex ratio, Antimalarial drugs

## Abstract

While significant advances have been made in understanding *Plasmodium falciparum* gametocyte biology and its relationship with malaria parasite transmission, the gametocyte sex ratio contribution to this process still remains a relevant research question. The present review discusses the biology of sex determination in *P. falciparum*, the underlying host and parasite factors, the sex specific susceptibility to drugs, the effect of sex ratio dynamics on malaria parasite transmission and the development of gametocyte sex specific diagnosis tools. Despite the inherent differences across several studies and approaches, the emerging picture highlights a potentially relevant contribution of the *P. falciparum* gametocyte sex ratio in the modulation of malaria parasite transmission. The increasing availability of molecular methods to measure gametocyte sex ratio will enable evaluation of important parameters, such as the impact of drug treatment on gametocyte sex ratio in vitro and in vivo as well as the changes of gametocyte sex ratios in natural infections, key steps towards elucidating how these parameters affect parasite infectiousness to the mosquito vectors.

## Background

Despite the recent public health effort, malaria remains one of the leading causes of death worldwide [1]. In the global effort aiming at disease elimination and marked by the use of effective anti-malarial drugs and insecticide-treated nets (ITNs), the *Plasmodium* gametocytes deserve a special attention, being the parasite blood stages responsible for human to mosquito parasite transmission [2]. Developing effective strategies to control malaria disease and block parasite transmission requires further understanding of the genetic determinants governing the parasite life cycle between vertebrate human hosts and female *Anopheles* mosquito vectors.

In the human host, a fraction of *Plasmodium falciparum* blood asexual stage produces sexual stages, male and female gametocytes. In this parasite, gametocytogenesis requires a comparatively more extended period (8–12 days) than in other human malaria parasite species [3, 4], in which five stages (I to V) of morphological development are routinely classified [5]. Gametocyte stages I to IV were reported to sequester in deep organs (bone marrow and spleen) while stage V gametocytes are found in the peripheral blood [6] where they take an additional 3 days to become infectious to mosquitoes [7–10]. Malaria parasite transmission requires the presence of both sexes of gametocytes for fertilization, eventually producing new parasite stages (the sporozoites) where genetic recombination has occurred and determines the genetic make-up of the parasite offspring [11].

During malaria infections, *P. falciparum* the rate of gametocyte production is influenced by several factors, both from the human host [4, 11] (haemoglobin level [12–14], host immunity [15, 16], anti-malarial drug treatment [17–19]) and from the parasite (genetic diversity of infection [20] or mixed infection [21, 22], asexual stage densities [23]). In several cases these studies have not concomitantly investigated whether and how these factors also modified the gametocyte sex ratio, which is defined as the ratio of the number of male gametocytes over female gametocytes and which is typically female biased in *Plasmodium* parasites [24]. Evidence of factors influencing the gametocyte sex ratio derives from controlled infections mainly on rodent parasites and from a few studies of *P. falciparum* natural infections or clinical trials.

The impact of anaemia on gametocyte sex ratio was experimentally shown in rodent and avian *Plasmodium* parasites [25–28] and in studies on *P. falciparum* infected children [14, 29]. A role of immunity was documented in *P. falciparum* infected children [30], whereas a modified gametocyte sex ratios was described upon drug treatment of *P. falciparum* infections [31, 32].

In vitro assays on *P. falciparum* gametocytes suggested that male gametocytes are more vulnerable to anti-malarial drugs than female gametocytes [33]. This effect has been recently investigated in vivo in clinical trials assessing the effect of combination drug treatments including primaquine or methylene blue. In both combination treatments these drugs were shown to inhibit parasite transmission and also to affect the gametocyte sex ratio, although in opposite directions, with methylene blue proposed to be more active on male gametocytes and primaquine on female gametocytes [34].

The present work aims to review studies on *P. falciparum* gametocyte sex determination, highlighting unanswered questions relevant to better understand consequences of sex ratio changes on the modulation of parasite transmission.^1^

### Sex determination in *Plasmodium falciparum*

During malaria infection, the parasite is faced with a resource allocation trade-off between investing in asexual proliferation for in-host survival or in sexual differentiation for between-host transmission [11].

The development into a male or a female gametocyte in *Plasmodium* is not determined by sex chromosomes as a single haploid parasite can generate both sexes [24, 35]. Functional investigations on the mechanism(s) of parasite commitment to sexual differentiation have shown that the expression of the PfAP2-G transcription factor is required for the transcriptional activation in stage I gametocytes of genes encoding early gametocyte specific proteins, such as Pfs16, PfMdv-1/peg3, Pfpeg4 and Pfg27, and for gametocyte production [3, 36–39]. Activation of *pfap2G* expression requires the release from epigenetic chromosomal silencing of the *pfap2G* locus orchestrated by the interaction of the nuclear proteins PfGDV-1 and HP1 [40, 41]. The first marker of gametocytogenesis in both male and female gametocytes is the expression and export in the red blood cell cytoplasm of the parasite protein *Pf* Gametocyte Exported Protein-5 (PfGEXP5) as early as 14 h post red blood cells invasion [42]. The observation that the expression of pfGEXP5 is PfAP2-G independent [42] and that a limited gametocyte transcriptional program is also initiated in a natural PfAP2-G mutant parasite line [43] altogether suggest that entry in and/or progression of sexual differentiation may involve additional, PfAP2-G independent, mechanism(s).

Independent investigations on *P. falciparum* commitment to the production of male and female gametocytes indicated that distinct subpopulations of schizonts produce a homogeneous progeny of either all male or all female gametocytes [44, 45]. This is consistent with previous evidence that individual *P. falciparum* schizonts are alternatively committed to either gametocyte production or to further asexual multiplication [46] and that, in transgenic pfAP2-G-tagged parasites, schizonts are distinguishable for containing either all PfAP2-G positive or all AP2-G negative merozoites [47, 48]. This mechanism of gametocyte sex commitment implies that *P. falciparum* modifies sex ratio by changing the proportion of schizonts committed to male or female gametocyte production. Considering also the long time required for *P. falciparum* gametocytes to be mature for fertilization, parasite sex ratio adjustment in response to any given factor appears to be a slow process, indeed appreciable over long infection times.

To partly correct this picture, recent reports showed in *Plasmodium berghei* and in *P. falciparum* that induction or stabilization of AP2-G expression early after merozoite invasion can redirect differentiation of the newly invaded parasite towards becoming a gametocyte [48, 49]. If this modulation also applied to sex determination and occurred in natural infections, this would introduce a slightly higher flexibility in the parasite adaptive mechanism to govern its sex ratio. In any case, the above investigations altogether indicate that the gametocyte sex is determined very early, and possibly at the same time, of the parasite commitment to sexual differentiation.

In *P. falciparum,* the morphological differences between male and female gametocytes appear as early as stage III [50], approximately 4 days post red blood cells invasion. ‘Omics’ analyses of purified female and male gametocytes marked by specific fluorescent reporters in *P. berghei* [51] and in *P. falciparum* [52] showed that sexual dimorphism is based on major differences in the transcriptomes and proteomes of the two sexes, which are responsible for the divergent physiology of male and female gametocytes geared to achieve gamete fertilization in the mosquito host and essential for life cycle progression [51, 52]. In *P. falciparum*, 247 out of 2110 proteins were found to be differently expressed between sexes, 206 in male and 41 in female gametocytes [52]. Proteins expressed exclusively in male gametocytes were mainly annotated as involved in genome replication and formation of eight motile gametes, whereas the female specific proteins were associated with metabolism, translation and organelle functions [51–53].

In some cases, proteins previously described as gametocyte specific were reported to have sex specific roles, such as Pfg377 [54], involved in the production of osmiophilic bodies [55], produced in female gametocytes since the stage III [56], the 6-cysteine domain protein Pfs47, expressed exclusively in female gametocytes from stage II [57], and the gamete surface protein Pfs25 [58], whose transcript has been the first marker used to quantify *P. falciparum* female gametocytes in reverse transcriptase quantitative PCR (qRT-PCR) [59]. This analysis also confirmed the male gametocyte specific expression of the Pfs230 protein, expressed in stages IV–V [60], whose transcript has been used to quantify male gametocytes in RT-PCR assays [59].

On the other hand, proteins expressed in both gametocyte sexes have been proposed to play a sex specific role in male gametocytes, such as Pfs48/45, expressed on the gametocyte surface from stage II [61], whose ablation reduced only male gamete ability to penetrate female gametes [57], or the regulatory protein *PfPuf2,* whose ablation showed a phenotype only in the differentiation of male gametocytes [62].

Of specific relevance for the ability of male and female gametes to produce and respond to external cues in order to fertilize and to produce a viable diploid mosquito stage, the above ‘omics’ analyses revealed, both in *P. berghei* and *P. falciparum,* a sex-specific distribution of signaling proteins such as kinases and phosphatases [51, 52]. Five protein kinases and ten phosphatases were identified as upregulated in *P. falciparum* female gametocytes, whereas ten kinases and three phosphatases were upregulated in male gametocytes [52]. In *P. berghei*, two kinases were identified to be female gametocyte specific whereas two kinases and four phosphatases were specific to male gametocytes [51].

### Host and parasite factors affecting *Plasmodium* gametocyte sex ratio

The female biased sex ratio in *Plasmodium* parasites [63–65] is intuitively explained as a way to optimize fertilization since a single male gametocyte produces eight flagellated microgametes, a feature particularly important when gametocyte densities become critically low [17]. The gametocyte sex ratio can nevertheless be affected by human as well as parasite factors through mechanisms that still need to be elucidated [25, 45, 66].

Several investigations proposed a role of human host factors, such as anaemia and immunity [11, 67–70] and of parasite factors such as asexual parasite density [4], parasite diversity [71] and competition, as well as the duration of malaria infection [12, 72–74]. Anaemia, a factor associated to malaria, was shown to affect parasite sex allocation not only through increased gametocyte production but also through a more male-biased sex ratio [25, 27], with the erythropoietin (Epo) hormone, an erythropoiesis inductor, being shown in rodent and avian malaria parasites to play a role in the male biased shift of sex ratio [25]. Anaemia was also described to prolong the survival of male gametocytes, favoring malaria transmission [69].

In addition to host factors involved in erythropoiesis and anaemia, the immune response developed against asexual parasites and gametocytes has been proposed to affect sex ratio as the proportion of male gametocytes was reported to increase in prolonged malaria infections [75]. This might be viewed as a parasite evolutionary strategy to increase proportion and total number of the cell type more vulnerable to antibody attack. Indeed, although antibodies against gametocyte surface antigens, acting only after gamete egress from the erythrocyte in the mosquito gut [76, 77], affect both sexes (e.g. by efficiently agglutinating gametes), they are particularly effective against the short lived, motile male gametes [76]. A higher rate of non-synonymous vs synonymous mutations was calculated in the coding sequences of male expressed genes, revealing that male gamete antigens undergo a comparatively faster adaptive evolution than other parasite antigens [78]. This may represent an additional, complementary strategy to protect male gametes from the mounting immune response.

One parasite factor affecting sex ratio is the competition between parasite clones [20, 30, 79]. It has been observed in multiclonal *P. falciparum* infections that each parasite clone produces a more male biased gametocyte population [30], indicating that competition between clones may favor a more even sex ratio, which was interpreted as a way to maximize each clone fertilization efficiency [30, 35, 80]. In human malaria infections, the presence of multiple genotypes may be explained by either subsequent infections or simultaneous acquisition of multiple clones from a single mosquito bite [81]. Understanding how the genetic complexity of co-infecting parasite clones and the subsequent changes in sex allocation strategies may influence parasite infectivity to mosquitoes is a major goal to elucidate malaria parasite transmission dynamics.

### Gametocyte sex specific susceptibility to antimalarial drugs

Anti-malarial drug treatments aim to cure malaria infections as rapidly, reliably and safely as possible [82] and are for this reason targeted to hit the asexual pathogenic forms of the parasite. However, anti-malarial drugs, including artemisinin derivatives, are generally less or not at all effective on gametocytes [83–85]. Treatment failure is associated to an increased gametocyte carriage and clinical observations revealed that chloroquine and sulfadoxine-pyrimethamine post treatment gametocytaemia can be considered as an early parasitological indicator of reduced drug sensitivity [19, 86, 87].

In the context of malaria elimination and eradication, the need of targeting gametocytes, in *P. falciparum* so far attacked only by primaquine, is widely recognized. *In vitro* assays testing a panel of anti-malarial drugs on *P. falciparum* and *P. berghei* gametocytes indicated that male gametocytes show a higher vulnerability than females to drug treatment [33], leading to suggest the strategy of targeting this minority fraction of more susceptible sexual stages, yet required for fertilization, to efficiently limit malaria parasite transmission [88].

This issue is now being addressed in *in vivo* settings. Reports evaluating gametocyte clearance by anti-malarial drugs and sex specific densities could not find significant changes in gametocyte sex ratio [30, 89]. On the other hand, two recent clinical trials studied the effect of anti-malarial drugs on gametocyte sex ratio in vivo and concluded that treatments produced in both instances a shift in the gametocyte sex ratio. One clinical trial was conducted in Kenya with 120 infected children from an area of moderate malaria transmission. RT-qPCR assays amplifying the female marker *pfs25* and the male marker *pfGMET* were used to measure the gametocyte sex ratio after a dihydroartemisinin-piperaquine treatment alone or combined with primaquine. In both cases male gametocytes appeared to be cleared more slowly than females, resulting in a male shifted sex ratio [32]. Another clinical trial was conducted in Mali on 80 infected males aged 5 to 55 from a hyperendemic, highly seasonal malarious region. The same assays were used to measure effect on gametocyte sex ratios of the addition of primaquine to a sulfadoxine-pyrimethamine and amodiaquine treatment and of the addition of methylene blue to a dihydroartemisinin-piperaquine treatment. The significant decrease in overall gametocyte density associated in particular to the treatment with methylene blue was in this case associated with a strongly reduced circulation time of male gametocytes and an increased female sex ratio [32, 34]. In this study, effect of the different drug regimens on parasite transmission was assessed by measuring the proportion of mosquitoes that became infected after feeding on blood samples of the trial participants taken before and after 2 and 7 days from treatment. Interestingly, a significant decrease of infection prevalence was observed already after 2 days of the treatments including primaquine and methylene blue, when however gametocyte density (by microscopy) and proportion of male and female gametocytes (by RT-qPCR assays) were not affected. This result, which confirmed previous observations on the activity of primaquine [85] was recently interpreted as an evidence of an early sterilizing effect on gametocytes of the two drugs, whose transmission-blocking effects precede gametocyte clearance [90]. As the latter assays measure density of male and female gametocytes amplifying sex specific transcripts, it is possible that the target mRNAs persist in morphologically detectable, but likely damaged or dead gametocytes, affecting the assay predictability of parasite viability. This suggests that evidences of gametocyte sex specific drug susceptibility have to be object of additional studies and of independent molecular and cell-based assays. This also calls for the urgent need of reliable molecular markers of gametocyte fitness, predictive of mosquito infectiousness, in whose absence xenodiagnosis remains of key importance to assess transmission-blocking properties of anti-malarials.

### Development of gametocyte sex specific diagnostic tools and application in investigating sex ratio dynamics

The identification of gametocyte carriers is important for determining the sources of infection in a community, but gametocyte presence and concentration are not linearly predictive of parasite infectiousness to mosquito. Measuring gametocyte sex ratio may, therefore, provide a different, possibly more predictive, parameter for mosquito infectivity to be used in improved control strategies. Early studies in endemic areas that included gametocyte sex ratio assessments have used laborious microscopy techniques [69, 89]. However, a critical issue is that identification and counts of male and female gametocytes relied upon inspection of Giemsa stained smears by conventional light microscopy and by sexing gametocytes by the pink or blue color of the cytoplasm of male and female gametocytes, respectively. Obviously, these measurements could be performed only in microscopy positive slides, with densities of at least ten gametocytes/µl [14, 30], and prevented measuring sex ratio in infections with submicroscopic gametocyte carriages [91, 92]. To overcome this problem, in addition to improving the molecular tools to detect parasitaemia and overall gametocytaemia [81, 93], methods have been developed in the past few years to specifically detect and quantify male and female gametocytes, based on the reverse transcription and polymerase chain reaction (RT-PCR) amplification of gametocyte sex specific transcripts.

The first study was the report by Schneider et al. [59], which used the *pfs25* and *pf230p* mRNAs as female and male markers, respectively, to be amplified in *P. falciparum* mature gametocytes. These genes were chosen due to their expression during late stage gametocytes and, as *pfs25* shows a limited polymorphism, this marker is currently used to quantify gametocytes from field isolates, also relying on the fact that female gametocytes are generally the more abundant sex [59, 94, 95]. The transcriptomics analysis of purified male and female gametocytes [52] provided additional sex specific transcripts as reagents in improved sex specific q-RT-PCR diagnostic assays. The *pf13* (PF3D7_1311100) and the *pfGK* (PF3D7_1351600) mRNAs were used as male and female gametocyte specific markers in a homogeneous SYBR-Green q-RT-PCR assay, utilized to measure gametocyte sex ratio in epidemiological samples from Burkina Faso [96]. A novel male gametocyte marker, *pfMGET* (*Plasmodium falciparum* male gametocyte-enriched transcript, PF3D7_1469900), was also described in a novel gametocyte sex specific RT-PCR assay [32]. To overcome the limitation that separate quantification of male and female gametocytes could bias the reliable determination of sex ratio, a novel multiplex assay was developed, using the above *pfMGET* mRNA and the female pfCCp4 (PF3D7_0903800) transcript. An important improvement in this assay, besides multiplexing, was that both transcripts are amplified from an intron containing region, which enables quality control of the amplification product and avoids DNAseI sample treatment [97].

### The effect of *Plasmodium* gametocytes sex ratio on malaria parasite transmission

Gametocyte sex ratio has been shown to be under strong selection pressure both as an adaptive response to a worsening blood environment for transmission and to the number of co-infecting clones in vertebrates [11, 28, 79]. However, the impact of sex ratio dynamics on the transmission success of *P. falciparum* is still far from being fully explained and represents a major challenge in translating sex ratio measurements into prediction of parasite transmissibility.

Some in vitro [98] and in vivo infections [65] studies have shown that a higher male sex ratio was more infectious to mosquitoes. As the gametocyte sex ratio becomes significantly male biased when gametocytes density is low [64, 99, 100], it is critical to elucidate the functional relationship between sex ratio and gametocytes density. In this respect, the analysis of gametocyte sex ratios and of the resulting mosquito infectivity data from 148 feeding experiments on naturally infected gametocyte carriers in Mali, Burkina Faso and Cameroon importantly showed that male gametocyte presence becomes critically important only at low gametocyte densities [101].

Integrating the information gained in epidemiological studies and clinical trials on microscopy-positive and sub-microscopic gametocyte densities and on gametocyte sex ratios using the recently developed q-RT-PCR techniques will be of key importance to investigate factors affecting malaria transmission at the population level.

## Conclusion

In recent years, the challenge to eliminate malaria focused on the control of malaria parasite transmission and highlighted the gametocytes and their biology as a crucial objects of investigation. The development of molecular tools based on gametocyte sex specific markers is increasingly pairing with novel relevant bioassays for the assessment of gametocyte functionality and viability [102]. Altogether these tools will enable us to understand if and how some anti-malarial drugs differentially affect mature male and female gametocytes in natural infections and in laboratory settings. The main challenge of these assays is to what extent they are and will be able to translate determination of gametocyte numbers into measurements of gametocyte fitness. This will be critical to confidently use gametocyte sex ratio to predict parasite infectiousness to mosquito, with an obvious impact on our ability to implement appropriate and effective measures to block parasite transmission.

## Abbreviations

RNA: ribonucleic acid
RT-PCR: reverse transcriptase-polymerase chain reaction
PQ: primaquine
PfGDV-1: *Plasmodium falciparum* gametocyte development 1
PfHP1: *Plasmodium falciparum* heterochromatin protein 1
*PfGK*: *Plasmodium falciparum*: glycerol kinase
*Pf*MGET: *Plasmodium falciparum* male gametocyte-enriched transcript
q-RT-PCR: quantitative real time PCR

## Footnotes

1 While this manuscript was in preparation, the topic of this review was covered by an excellent review by Tadesse et al. [103].
